# Pediatric Appendicitis Score and Ultrasound in children with Acute Appendicitis: An Observational Study

**DOI:** 10.31729/jnma.8889

**Published:** 2025-02-28

**Authors:** Suyog Simkhada, Bijay Thapa, Anupama Thapa Basnet, Sijan Karki

**Affiliations:** 1 Department of Pediatrics Surgery, Kanti Children Hospital, Maharajgunj, Kathmandu, Nepal; 2 Bir Hospital, Mahaboudha, Kathmandu, Nepal

**Keywords:** *acute appendicitis*, *computed tomography scan*, *histopathological examination*, *pediatric appendicitis score*, *ultrasonography*

## Abstract

**Introduction:**

Acute appendicitis is a common surgical emergency encountered in pediatric population. Differential diagnosis in this age group present with similar signs and symptoms. Clinical scoring system and Ultrasound imaging are helpful in diagnosing acute appendicitis and ruling out its differential diagnosis. This study was done to assess the findings of presence or absence of appendicitis in children based on the Pediatric Appendicitis Score (PAS) and ultrasound scan.

**Methods:**

An observational cross-section study was done from November 2023 to May 2024, in a tertiary level hospital, Kathmandu. All children between the ages of five and 14 years undergoing surgery for the provisional diagnosis of acute appendicitis were included in the study. The provisional diagnosis was based on a Pediatric appendicitis score and ultrasound. The diagnosis was confirmed by histopathology. Ethical approval was taken from Institutional Review Committee (Reference number: 811/2080/81).

**Results:**

A total of 50 children were included during the study period. A pediatric appendicitis score of more than seven, which is considered a high risk, was observed in 43 (86%) patients, and a score between four and six, which was considered an intermediate risk, was observed in 6 (12%) patients Similarly, 39 (78%) of the patient had appendix diameter more than six mm and 10 (20%) had less than six in ultrasound measurement.

**Conclusions:**

Both Pediatric Appendicitis Score and Ultrasonography can be used to diagnose acute appendicitis in children.

## INTRODUCTION

Acute appendicitis, an inflammatory condition of the appendix, results from ischemic mucosal damage, bacterial overgrowth, and luminal obstruction, often due to lymphoid hyperplasia or fecolith.^1,2^ Globally, its incidence is 1-2 per 10,000 children under 4 years, rising to 25 per 10,000 by the second decade, with higher rates in regions like India and Bhutan (51.5 per 10,000).^3-5^

Timely diagnosis is challenging due to overlapping symptoms with nonsurgical diseases.^6^ The Pediatric Appendicitis Score (PAS), based on clinical presentation, physical symptoms, and laboratory testing, aids diagnosis in children.^7-10^ Ultrasonography, preferred over CT scans for its safety and efficiency, is a key diagnostic tool.^7,11,12^

There have however not been any similar previous study in our population setting. So, to fullfill the knowledge gap this study was done to see that the total clinical scoring by the PAS along with ultrasonography measured diameter of the appendix can confirm the histopathological diagnosis of acute appendicitis. This study also help us find out the demography and clinical presentations of acute appendicitis in our population of children.

## METHODOLOGY

This was a observational cross-section study done from a period of November 2023 to May 2024, after ethical approval from Institutional Review Committee (IRC) of National Academy of Medical Sciences (NAMS) (Reference number: 811/2080/81). Children suspected of having acute appendicitis were admitted to the department of Pediatric Surgery at Kanti Children's Hospital. The total of 50 patients over the period of 6 months whose PAS score and USG along with histopathology were observed and recorded.

Primary and secondary data were collected through interview and examination of the patient, using a semi-structured preformed proforma after approval from review board. Symptoms and signs were assessed, and PAS scoring was done and recorded in the proforma accordingly.

The Pediatric Appendicitis Score (PAS) is a clinical scoring system designed to aid in the diagnosis of acute appendicitis in children. It comprises eight parameters, each assigned a specific score: anorexia (1), nausea (1), fever (1), migration of pain (1), tenderness in the right lower quadrant (2), cough/percussion/hop tenderness (2), leucocytosis (1), and neutrophilia (1), with a total possible score of 10.^13,14^ The interpretation of the PAS is categorized into three risk levels: low risk (PAS < 3), moderate/intermediate risk (PAS 4-6), and high risk (PAS > 7). This scoring system helps clinicians stratify the likelihood of appendicitis and guide further diagnostic and management decisions.

Blood investigations were sent to look for leukocyte and neutrophil counts, which are the components of the Pediatric Appendicitis Score. A leukocyte count of >10,000/mm^3^ and a neutrophil percentage of >75% were considered positive. Findings of anorexia, fever (>38°C), nausea, migration of pain, tenderness in right lower quadrant and cough/hop tenderness was assessed and scoring was given accordingly.

Subsequently, an ultrasound scan of abdomen and pelvis was performed in the radiology department by a single radiologist. The measurement of the diameter of the appendix was documented. The ultrasound machine used was Samsung SONOACE R7, equipped with a linear probe, model number L5-12/50. Non compressible appendix diameter of >6 mm (outer to outer thickness), was considered a positive radiological finding suggestive of inflammation as per reference value of diameter taken from one of the studies.^15^ Data was recorded on the basis of ultrasound finding of presence or absence of appendicitis according to diameter measured.

After the evaluation and recording of PAS and ultrasonography in all the 50 patients, counselling for surgery was done and consent was obtained from the parents accordingly.

Presence of appendicular lump including other pathology, patient below 5 years and above 15 years of age, and history of previous appendectomy were not taken into the study.

The decision to operate was made by the attending pediatric surgeon. Then the children underwent open appendectomy. The specimen were collected and sent for histopathological examination as standard protocol. Upon follow-up, the histopathology reports were reviewed to confirm the pathological diagnosis. Histopathology examination has been found to be a confirmatory test that may show presence of ulceration, diffuse infiltration of polymorphonuclear leukocytes, lymphocytes or mononuclear cells to confirm acute appendicitis.^16^ The histopathological diagnosis in our study was then recorded along with the initial USG findings and PAS scoring system. Data were entered in Microsoft excel and analysed by IBM SPSS version 24.

## RESULTS

Total of 50 children who presented with suspected acute appendicitis whose PAS and ultrasonography were evaluated and recorded, underwent open appendectomy along with Histopathological Examination (HPE) findings. There were four (8%) female and 46 (92%) male children (Table 1).

The age of children ranged from 5 years to 14 years with the mean age of 9.25±2.75 yrs. Among individuals with a PAS score of 3-6, 6(12%) had features of inflammation in HPE. In those with a PAS score greater than 7, 43(86%) had features of inflammation in HPE (Table 1).

**Table 1:** PAS score and HPE findings in children with acute appendicitis (n=50).

PAS score	Feature of Inflammation in (HPE)	No feature of inflammation in (HPE)	Total n(%)
PAS (3-6)	6(12%)	1(2%)	7(14%)
PAS >7	43(86%)	-	43(86%)
Total	49(98%)	1(2%)	50(100%)

PAS: Pediatric Appendicitis Score; HPE: Histopathological Examination

Among individuals with an appendix diameter of less than 6 mm on USG, 10(20%) had features of inflammation in HPE. In those with a diameter greater than 6 mm, 39(78%) had features of inflammation (Table 2).

**Table 2:** USG and HPE findings in children with acute appendicitis (n=50)

	Diameter of appendix (mm)	Feature of Inflammation in (HPE)	No feature of inflammation in (HPE)	Total n(%)
USG feature suggestive	Absent (<6 mm)	10 (20%)	1(2%)	11(22%)
of Appendicitis	Present (>6 mm)	39(78%)	0(0%)	39(78%)
Total		49(98%)	1(2%)	50(100%)

USG: Ultasound; HPE: Histopathological Examination

## DISCUSSION

Although its the incidence of acute appendicitis is lower in infants and children less than four years, it continues to be one of the common causes of abdominal pain in children with occurence in regional and local regions like India, Bhutan with 51.5 per 10,000 population.^3,4^ 50 of patients were included in our study similar to one of the study done locally in our country where total of 70 children with acute appendicitis were studied the only difference being the time interval 6 month in our study whereas it was 1 year in the above study.^17^ Studies done globally and regionally have shown incidences are much higher during teenage years 10-19 years and even as below 5 years.^3,18^ This is similar to our study where the age range was 5-14 years with the mean age of children was 9.25±2.75 yrs. However none of the children in our study were below 5 years of age may be due to the local population of ours where peak appendicitis is during 5-14 years of age. Studies have shown that male is more frequently affected with the lifetime risk of 8.6% compared to 6.7% in females.^19^ Amongst the 50 children in our study 46 (92.00%) were males and four (8.00%) were females showing the preponderance of male children. A similar study showed majority of male of 61% and female 39% in total of 60 children which is similar to our finding^14^

Over the time of various research the pediatric appendicitis scoring system has been studied and most study suggesting PAS <3 as low risk with less likelihood of appendicitis, PAS(4-6) intermediate risk and PAS score >7 high risk with high likelihood of appendicitis.^13, 14^ The PAS in our study was categorized similarly. However there were no patients with low risk score < 3 in our study. A similar study was done in 60 children out of which 10 patients had PAS <3 and 20 (33%) had their PAS >7 and their biopsy confirmed appendicitis suggesting that PAS >7 is strongly indicative of appendicitis requiring surgery.^14^ In our study all 43 (86.00%) of the patients with high-risk score PAS >7 had histopathology confirmed diagnosis of appendicitis. In the same study 30 of patients had intermediate risk PAS (4-6) and out of which 10 (16%) were operated and after surgery all were confirmed to have appendicitis in biopsy.^14^ In our case for intermediate risk PAS (4-6), six (12.00%) of the patients were confirmed to have acute appendicitis with features of inflammation and only one (2.00%) case did not have features of inflammation on the histopathological examination. Iftikhar el al. studied the PAS and concluded PAS to be a superior scoring system with a cut of value of 7 suggesting PAS >7 is strongly indicative of appendicitis in children.^20^ In another study out of 104 patients low risk probability PAS (score <4) was seen in 8.7% children; 43% had equivocal PAS (score 4-6) and 49% had high risk PAS (score >6), after surgery biopsy proven appendicitis was seen in 43 (97.7%) and 50 (98%) children with equivocal and high-risk PAS, respectively which is similar to our findings.^21^

In our study the patients underwent Ultrasonography scan to look for features of inflammation according to the diameter of appendix. Although CT may give more accuracy but due to exposure to radiation and contrast material the preferred investigation is an ultrasonography.^22,23^ One of the strong indicator and direct sign of inflamed appendix is diameter with >6 mm from few studies.^15,22^ In our study all the 39 (78.00%) cases with strong USG feature of appendicitis >6 mm was confirmed with appendicitis on histopathology and none of them had non-inflammatory feature on histopathology. So, with USG diameter of >6 mm all 39 (78.00%) cases had confirmed appendicitis. Similarly, our study found out total 11 (22.00%) cases who had absence of features of appendicitis on USG <6 mm diameter, out of which 10 (20.00%) of the cases were found to have acute appendicitis with features of inflammation on histopathology and only one (2.00%) case did not have any inflammatory feature on histopathological examination. A similar finding by Elahifar MA where out of 200 patients, one hundred sixty-six patients (83%) were diagnosed as acute appendicitis on pathology, and 34 (17%) were diagnosed differently.^24^ 157 of patients underwent US, eighty two of this patients diagnosed as acute appendicitis on US examinations and in 78 of them were also reported as acute appendicitis on confirmatory histopathological examination concluding that a positive ultrasound is indicative of appendicitis, but a negative ultrasound does not rule out appendicitis. Hajalioghli et al. measured diameter of appendix in two groups, first group 37 cases (30.6%) with appendix diameter equal or <6 mm, compressible with (avg. diameter 4.5±1.06mm) suggesting risk appendicitis and second group 49 (40.5%) cases with strong suggested appendicitis with noncompressible appendix and diameter of >6 mm ( avg. diameter 8.5±3.01mm) where 4 patients underwent appendectomy in group first, out of which 2 cases had positive pathologic findings of appendicitis, while 2 did not have pathologic appendicitis and 47 patients in group second underwent appendectomy, out of which 46 patients had a positive HPE for appendicitis and 1 patient had no pathologic appendicitis in HPE, ^25^ which is similar to our finding.

We could only study upon 50 patients large population might have given more results. USG is an operator dependent tool and the result can vary accordingly.

## CONCLUSIONS

The study reports the prevalence of acute appendicitis using the Pediatric Appendicitis Score (PAS) and ultrasonography (USG) findings. A PAS >7 and an appendix diameter >6 mm on USG were commonly observed in confirmed cases. These results highlight the utility of PAS and USG in assessing suspected appendicitis in children.
